# Gender-related and Age-related Disparities in Prevalence of the Cardiovascular-Kidney-Metabolic Syndrome Among US Adults From 1999-2020: An Analysis of the NHANES Survey

**DOI:** 10.1016/j.xkme.2025.101234

**Published:** 2025-12-26

**Authors:** Zhejia Tian, Samira Soltani, Johann Bauersachs, Kai M. Schmidt-Ott, Anette Melk, Bernhard M.W. Schmidt

**Affiliations:** 1 Department of Nephrology and Hypertension, Hannover Medical School, Hannover, Germany; 2 Department of Cardiology and Angiology, Hannover Medical School, Hannover, Germany; 3 Department of Pediatric Kidney, Liver and Metabolic Diseases, Hannover Medical School, Hannover, Germany

**Keywords:** Cardiovascular-kidney-metabolic syndrome, cardiovascular disease, chronic kidney disease, epidemiology, metabolic risks, women health

## Abstract

**Rationale & Objectives:**

The cardiovascular-kidney-metabolic (CKM) syndrome is defined as the intricate interplay among metabolic risks, chronic kidney disease (CKD) and the cardiovascular system. The deteriorating CKM syndrome contributes to untimely morbidity and mortality. We aim to characterize gender- and age-related disparities in the prevalence of CKM syndrome over the last 2 decades.

**Study Design:**

A cross-sectional population-based survey.

**Setting & Participants:**

A total of 32,848 US adults participating in the NHANES survey from 1999 to 2020.

**Exposures:**

Gender, age (18-44, 45-64, and ≥65), and period (1999-2002, 2003-3008, 2009-2014, and 2015-2020).

**Outcomes:**

Prevalence of CKM stages.

**Analytical approach:**

Sample weights and Taylor series linearization method were applied to estimate prevalence and standard errors representative of the noninstitutionalized US adult population. For trend analysis across cycles, survey-weighted logistic regression was employed.

**Results:**

Young women aged < 45 years were classified more often, but with decreasing prevalence, in stages without CKM defining factors (22.7% of women vs 13.5% of men) and more often in stages with cardiovascular organ damage (13.4% of women vs 6.5% of men). Elderly women were increasingly classified in stages with cardiovascular organ damage over the last 20 years, reaching the same prevalence as men in the most recent period (25.3 % [95% CI, 20.0 %-30.6 %] of women vs 30.5 [95% CI, 25.7-35.3%] of men aged > 65 years).

**Limitations:**

NHANES data allow for assessing CKM stages with cardiovascular organ damage mainly based on self-reporting during interviews.

**Conclusions:**

We demonstrate an increasing proportion of women in advanced CKM stages over the last 20 years. Whereas the overrepresentation of younger women in the low-risk stages almost disappeared, elderly women in the last period showed almost the same risk of being in stages with cardiovascular organ damage as elderly men. Our analysis highlights an urgent need of preventive measures especially tailored to women.

## Plain-language Summary

Cardiovascular-kidney-metabolic (CKM) syndrome describes the combined impact of heart, kidney, and metabolic health on overall well-being. We examined its prevalence among US adult population and explored differences between women and men across different age groups, using data from over 32,000 adults collected from 1999 to 2020. The results showed that younger women under 45 years were previously more likely to be in the low-risk group; however, this advantage has declined over the past 20 years. Among older adults (over 65 years), women had a comparable risk to men for organ damage associated with cardiovascular-kidney-metabolic syndrome. Our study highlights that there is an urgent need for prevention strategies especially tailored to women.

The cardiovascular-kidney-metabolic (CKM) syndrome is a recently defined long-term health condition characterized as a systemic disorder that signifies intricate interactions among metabolic risk factors, chronic kidney disease (CKD), and the cardiovascular system. It transcends the simple sum of its components, leading to multiorgan dysfunction and increased adverse cardiovascular outcomes.^1^^,^^2^ Four stages of CKM syndrome were outlined: stage 0, characterized by the absence of CKM risk factors; stage 1, marked by excess or dysfunctional adiposity; stage 2, involving metabolic risk factors and/or CKD (Table S1); and stages 3-4, defined by subclinical (stage 3) or clinical (stage 4) cardiovascular disease (CVD) alongside CKM defining factors^1^^,^^2^ (Table S2).

We and others have determined the prevalence of the different CKM stages based on NHANES data.^3^^,^^4^ Both analyses consistently showed a very high prevalence of CKM syndrome among the adult population. Only fewer than 10% of US adults did not present any CKM risk factors (stage 0). Therefore, to optimize CKM health management, additional information is necessary to tailor individualized preventive measures.

Over the past decades, it has been well established that cardiovascular health and risk factors are influenced by both gender and age, with their distribution and impact differing accordingly.^5^^,^^6^ Whereas women are relatively protected from cardiovascular disease until menopause for various reasons, like hormones, immunological differences, and genetic and epigenetic variations despite counteracting socioeconomic and psychological influence,^7^ they catch up during later live, when the latter becomes more prominent. Therefore, it is reasonable to assume that CKM risk factors also vary with gender and age.

Using data from the National Health and Nutrition Examination Survey (NHANES), this analysis was intended to delineate the gender- and age-related disparities in the prevalence of the different CKM syndrome stages as well as the gender-specific and age-specific importance of the various components of the CKM syndrome. As NHANES recorded the variable as gender, we used this term throughout our study, while acknowledging that both sex-related and gender-related factors influence the observed disparities.

## Methods

### Data Source and Study Population

The NHANES has been conducted continuously in 2-year cycles since 1999. It employs a cross-sectional survey, utilizing a complex, multistage, probability sampling design. The survey is representative for the noninstitutionalized, civil population of the United States. Written informed content was obtained from all survey participants, and the study procedures receive approval from the National Center for Health Statistics Research ethics review board. The Hannover Medical School institutional review board exempted the present study because the individual data remained deidentified. We followed the Strengthening the Reporting of Observational Studies in Epidemiology reporting guideline throughout our study.^8^

We included nonpregnant participants aged 18 year and older from 10 NHANES cycles starting with the 1999-2000 cycle until 2017-March 2020 cycle in our analysis. We used the 1999-2018 NHANES survey cycles for long-term all-cause and cardiovascular mortality analyses, as these are the only cycles linked to the US National Death Index. All participants should possess sufficient information to determine CVD based on self-report. All survey cycles were applied to evaluate the trends in the prevalence of CKM Stage 0-4 and were combined to analyze the overall prevalence.

### Data Collection

Demographic information was collected through household questionnaires. In NHANES, the gender variable is collected during the household interview and most likely reflects self-reported gender rather than sex at birth, but it is available only as a binary measure.

Data of body mass index, waist circumference, and blood pressure were extracted from clinical examination data. Standardized blood pressure measurements were performed: three consecutive measurements were taken at one-minute intervals, and the average of the last two measurements was applied to our analysis.^9^ Hypertension was defined by either elevated blood pressure according to the 2017 AHA guideline^10^ or the use of antihypertensive medication.

The CKD is classified by estimated glomerular filtration rate and albuminuria according to Kidney Disease: Improving Global Outcomes 2024 Guidelines as outlined in Table S1.^11^ Urinary albumin to creatinine ratio was extracted directly from medical examination data or was calculated from urinary albumin and urinary creatinine. We calculated estimate glomerular filtration rate using the 2021 race-free and ethnicity-free chronic kidney disease epidemiology collaboration creatinine equation.^12^

For lipid profiles, we considered medication use based on self-report and laboratory examination. The 2018 AHA guideline recommendations were applied to define the normal lipid condition.^13^

We evaluated diabetes conditions using self-reported information on glucose-lowering therapy, glycated hemoglobin, and fasting serum glucose. The presence of diabetes or prediabetes was defined based on diagnostic tests outlined in the 2023 ADA guidelines.^14^

### Stage Definition

We first applied a selection strategy consistent with the definitions of CKM syndrome stage 0-2^1^ (Table S2) to identify individuals meeting the criteria for stage 0-2 in each survey cycle and in overall combined cycles. Stages 3 and 4 were defined by self-assessment given in interviews. Because these data are not as precise as those defining stages 0-2, we combined stage 3 and stage 4 into stage 3-4. During the selection process, we identified a specific subgroup that remained undefined in the CKM system despite complete data. This included participants with isolated abnormal high-density lipoprotein-cholesterol or low-density lipoprotein-cholesterol. These participants fulfill neither the definition of stage 0 (requiring a normal lipid profile) nor of stage 1, confining cardiovascular risk that is not defined using CKM criteria. We designated this stage as stage X: risk that is not defined in the CKM system and categorized the risk of these participants between stage 0 (no risk factors) and stage 1 (excess or dysfunctional adiposity).

### Statistical Analysis

We accounted for the complex survey design factors for NHANES, such as sample weights, clustering, and stratification, as specified in the National Center for Health Statistic analytic guideline.^15^ In all analyses, morning fasting subsample weights and Taylor series linearization method were applied to estimate prevalence and standard errors representative of the noninstitutionalized US adult population.^16^^,^^17^ Each prevalence estimate was reported with a corresponding 95% confidence interval (95% CI). Chi-squared tests were used to compare categorical variables. For trends analysis across cycles, survey-weighted logistic regression was employed, with survey cycle as a continuous variable. To compare the 15-year cumulative incidences of all-cause and cardiovascular mortality between women and men across different age groups, time-to-event data were evaluated using a Kaplan-Meier-based approach and Cox proportional hazards regression models. Models were adjusted for sociodemographic factors, smoking status, and cycle years, variables not included in the CKM syndrome stage definition. 2-sided *P* < 0.05 was considered statistically significant.

Sensitivity analyses were conducted to examine the influence of nonresponse. There was some missingness in the data, which did not exceed ten percent in any of the parameters. We first adjusted sample weights with the adjustment cell method.^18^ Moreover, multivariate multiple imputation by chained equations with 5 imputations was performed to address missing data.^19^ All analyses were conducted using R version 4.3.2.

## Results

### Study Population Characteristics

Over these 10 survey cycles, 32,848 participants aged 18 years and older were included in our final study. These were representative for the noninstitutionalized US inhabitants (N = 215,480,397) (Table 1). Of all participants, mean ± SE age was 47.3 ± 0.2, 51.3% were female, and 48.7% were male. Participants aged between 18 and 44 years old constituted 46,3% of the total, with 35.6% aged between 45 and 64 years. About 18.1% were aged 65 years and older.

**Table 1:** Weighted Prevalence of CKM Syndrome Stages and Characteristics by Gender and Age in US Adult Population

	Overall	Women	Men
**Unweighted N**	32,848	16,851	15,997
**Weighted population**	215,480,397	110,443,571	105,036,825
	100%	51.3% (50.6-51.9)	48.7% (48.1-49.4)
**Weighted prevalence % (95% CI)**			
**Participants in stage 0 (% of US adults)**	7.9 (7.4-8.5)		
**Weighted prevalence of participants in stage 0 by age group and gender group**			
	100	64.0 (61.1-66.9)	36.0 (33.1-38.9)
Age 18-44	80.8 (78.1-83.5)	50.3 (47.1-53.5)	30.5 (27.6-33.4)
Age 45-64	17.0 (14.4-19.6)	12.3 (10.1-14.5)	4.7 (3.3-6.1)
Age **≥65**	2.2 (1.3-3.0)	1.4 (0.7-2.1)	0.8 (0.3-1.3)
**Participants in stage X (% of US adults)**	2.7 (2.4-3.0)		
**Weighted prevalence of participants in stage X by age- and gender group**			
	100.0	61.4 (56.3-66.5)	38.6 (33.5-43.7)
Age 18-44	70.9 (65.5-76.3)	42.6 (37.3-47.9)	28.3 (23.2-33.4)
Age 45-64	25.7 (20.6-30.8)	16.4 (12.5-20.3)	9.2 (5.7-12.7)
Age **≥65**	3.5 (1.8-5.2)	2.4 (0.9-3.9)	1.1 (0.2-2.0)
**Participants in stage 1 (% of US adults)**	18.3 (17.6-19.1)		
**Weighted prevalence of participants in stage 1 by age group and gender group**			
	100	48.8 (46.7-50.8)	51.2 (49.2-53.3)
Age 18-44	63.4 (61.1-65.6)	29.5 (27.8-31.3)	33.8 (31.9-35.7)
Age 45-64	30.6 (28.4-32.7)	16.2 (14.5-18.0)	14.3 (12.9-15.7)
Age **≥65**	6.1 (5.1-7.1)	3.0 (2.3-3.7)	3.1 (2.4-3.8)
**Participants in stage 2 (% of US adults)**	56.6 (55.6-57.5)		
**Weighted prevalence of participants in stage 2 by age group and gender group**			
	100	49.3 (48.3-50.2)	50.7 (49.8-51.7)
Age 18-44	38.5 (37.2-39.9)	16.0 (15.3-16.8)	22.5 (21.5-23.5)
Age 45-64	41.1 (39.9-42.3)	20.9 (20.0-21.8)	20.2 (19.3-21.1)
Age **≥65**	20.4 (19.5-21.2)	12.3 (11.7-13.0)	8.1 (7.5-8.6)
**Participants in stage 3/4 (% US adults)**	14.5 (13.9-15.1)		
**Weighted prevalence of participants in stage 3/4 by age group and gender group**			
	100.0	53.3 (51.2-55.4)	46.7 (44.6-48.8)
Age 18-44	31.6 (29.5-33.7)	21.0 (19.2-22.8)	10.6 (9.3-11.9)
Age 45-64	32.3 (30.4-34.2)	15.2 (13.7-16.7)	17.1 (15.5-18.7)
Age **≥65**	36.1 (34.1-38.1)	17.1 (15.5-18.7)	19.0 (17.5-20.5)

*Note:* Prevalences are presented as estimates with 95% confidence intervals. The percentages in the first row of each section refer to all US adults and add up to 100%. The percentages in the second part of each section refer to the population classified into the respective stage. Stage 0, population without any CKM risk factors; stage X, population with risk (isolated abnormal HDL-cholesterol or LDL-cholesterol that is not defined in the CKM system); stage 1, population with excess or dysfunctional adiposity without subclinical or clinical CVD; stage 2, population with metabolic risk factors or moderate-risk to high-risk CKD without subclinical or clinical CVD; stage 3 and 4, population with subclinical or clinical CVD.

Abbreviations: CKD, chronic kidney disease; CKM, cardiovascular-kidney-metabolic; CVD, cardiovascular disease; HDL, high density lipoprotein; LDL, low density lipoprotein.

### Prevalence of CKM Syndrome Stages and Characteristics by Gender and Age

**Figure 1:**
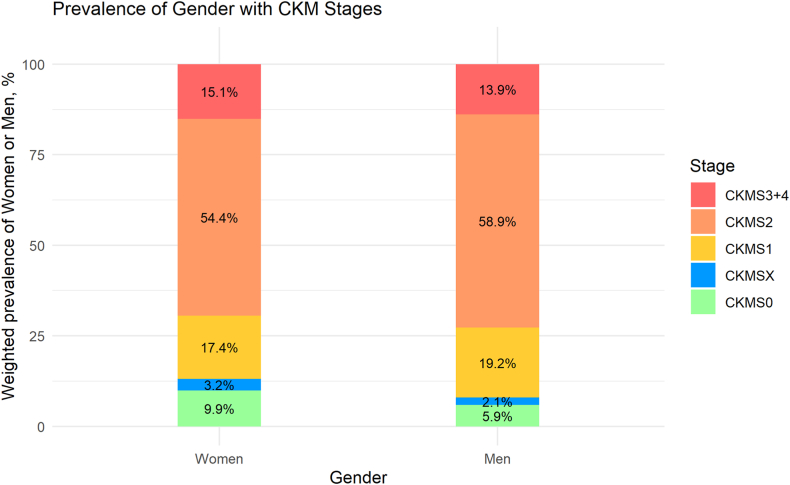
Gender-stratified prevalence in CKM syndrome stages 0-4. Weighted prevalence of CKM stage 0-4 of noninstitutionalized US adult women or men. CKM, cardiovascular-kidney-metabolic syndrome; S0, stage 0 without any CKM risk factors; SX, stage X with risk (isolated abnormal HDL-cholesterol or LDL-cholesterol that is not defined in the CKM system); S1, stage 1 with excess or dysfunctional adiposity without subclinical or clinical CVD; S2, stage 2 with metabolic risk factors or moderate- to high-risk CKD without subclinical or clinical CVD; S3-4, stage 3 and 4 with subclinical or clinical CVD.

Overall, 13.1% of women and only 8% of men were classified as having a CKM syndrome stage without CKM defining factors. About 17.4% of the female participants versus 19.2% of the male participants exhibited stage 1 CKM syndrome. More than 50% of each gender (54.4% of women vs 58.9% of men) demonstrated stage 2. Finally, 15.1% of women and 13.9% of men were assigned to stage 3-4 (Fig 1).

**Figure 2:**
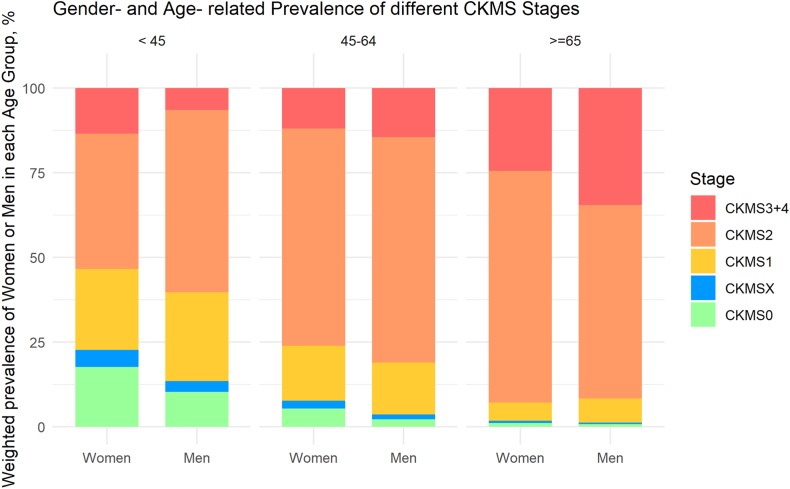
Age-stratified and gender-stratified prevalence in CKM syndrome stages 0-4. Weighted prevalence of CKM syndrome stages 0-4 in noninstitutionalized US adult women or men in different age groups (18-44, 45-64, and 65 and older). Women under 45 years: stage 0 17.7%, stage X 5.0%, stage 1 23.9%, stage 2 40.0%, and stage 3-4 13.4%; men under 45 years: stage 0 10.3%, stage X 3.2%, stage 1 26.2%, stage 2 53.8%, and stage 3-4 6.5%; women between 45-64 years: stage 0 5.3%, stage X 2.4%, stage 1 16.1%, stage 2 64.3%, and stage 3-4 11.9%; men between 45-64 years: stage 0 2.2%, stage X 1.5%, stage 1 15.3%, stage 2 66.6%, and stage 3-4 14.4%; women over 65 years: stage 0 1.1%, stage X 0.6%, stage 1 5.4%, stage 2 68.5%, and stage 3-4 24.4%; and men over 65 years: stage 0 0.8%, stage X 0.4%, stage 1 7.1%, stage 2 57.2%, and stage 3-4 34.5%. CKM, cardiovascular-kidney-metabolic syndrome; S0, stage 0 without any CKM risk factors; SX, stage X with risk (isolated abnormal HDL-cholesterol or LDL-cholesterol that is not defined in the CKM system); S1, stage 1 with excess or dysfunctional adiposity without subclinical or clinical CVD; S2, stage 2 with metabolic risk factors and/or moderate- to high-risk CKD without subclinical or clinical CVD; S3-4, stage 3 and 4 with subclinical or clinical CVD.

As summarized in Fig 2 and Table 1, as age increased, the prevalence of lower stages decreased, and the prevalence of the higher stages rose. In stages 0 and X, women were overrepresented, comprising about two-thirds of the participants in these groups. This difference between women and men remained significant in age groups of 18-44 years and 45-64 years. However, among elderly participants, this trend was no longer pronounced.

In stage 1, the gender distribution was almost even with men being slightly predominant in the younger age group (18-44 years): 33.8% of US adults in stage 1 (95% CI, 31.9-35.7) versus 29.5% (95% CI, 27.8-31.3).

At a younger age, more men were categorized into stage 2 because of metabolic risk factors or CKD: 22.5% of US adults in stage 2 (95% CI, 21.5-23.5) versus 16% (95% CI, 15.3-16.8). Conversely, this pattern shifted with increasing age, as a higher proportion of women aged 65 years and older were observed in stage 2: 12.3% of US adults in stage 2 (95% CI, 11.7-13) versus 8.1% (95% CI, 7.5-8.6).

Women were notably more prevalent in stage 3-4 compared with men, constituting 53.3% of US adults in these stages (95% CI, 51.2-55.4) versus 46.7% (95% CI, 44.6-48.8). This disparity was significant only in the 18-44 age group, 21.8% (95% CI, 19.2-22.8) versus 10.6% (95% CI, 9.3-11.9) of US adults in stage 3-4.

### Trends in Prevalence of CKM Stages by Gender and Age

**Figure 3:**
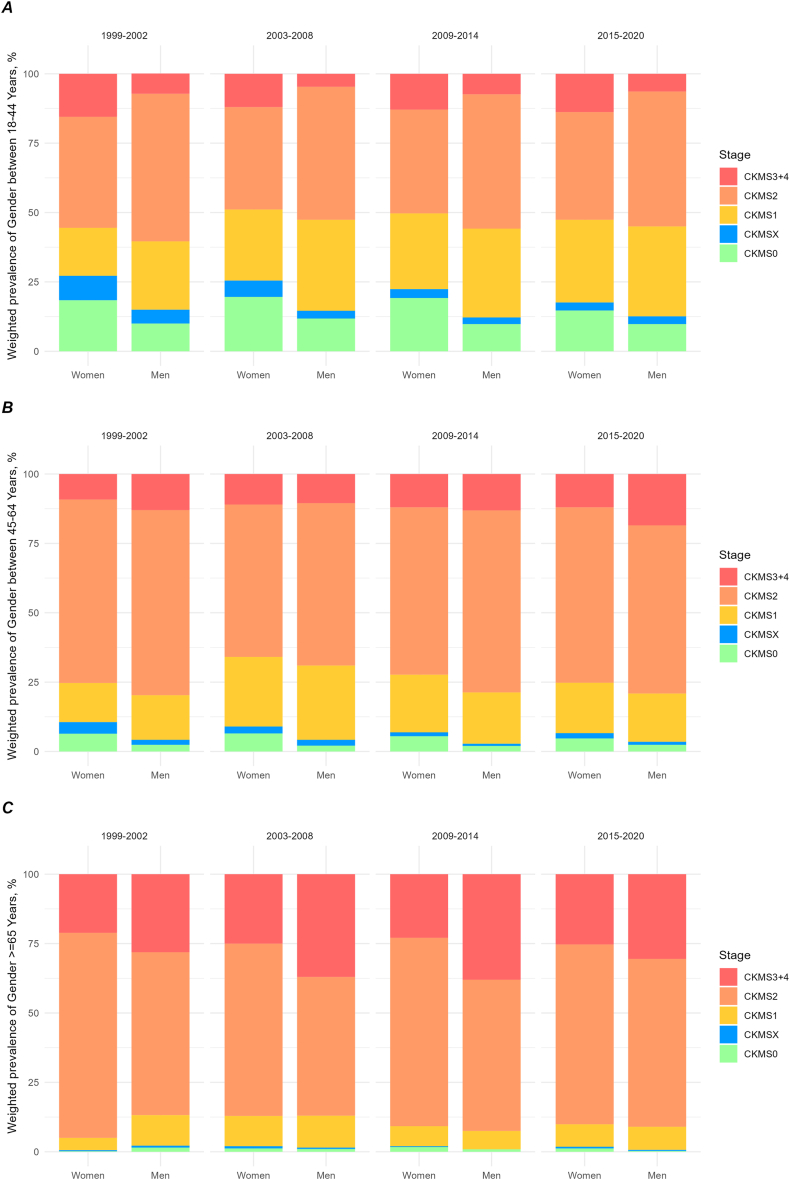
Temporal trends in gender-stratified and age-stratified prevalence of CKM syndrome stages 0-4. Trend analysis for weighted prevalence of CKM syndrome stages 0-4 in noninstitutionalized US adult women or men between 18-44 (A); trend analysis for weighted prevalence of CKM syndrome stages 0-4 in noninstitutionalized US adult women or men between 45-64 (B); and trend analysis for weighted prevalence of CKM syndrome stages 0-4 in noninstitutionalized US adult women or men aged 65 and older (C). CKM, cardiovascular-kidney-metabolic syndrome; S0, stage 0 without any CKM risk factors; SX, stage X with risk (isolated abnormal HDL-cholesterol or LDL-cholesterol that is not defined in the CKM system); S1, stage 1 with excess or dysfunctional adiposity without subclinical or clinical CVD; S2, stage 2 with metabolic risk factors and/or moderate- to high-risk CKD without subclinical or clinical CVD; S3-4, stage 3 and 4 with subclinical or clinical CVD.

The prevalence of US adult population below CKM stage 1 decreased from 13.2% in 1999-2002 to 8.5% in 2015-2020 (*P* for trend < 0.001) (Fig 3; Table 2). This declining trend was more pronounced among women, with a reduction from 8.5% to 5.2% of total US adults (*P* for trend < 0.001). In contrast, men experienced a smaller yet significant decrease from 4.7% to 3.5% of US adults (*P* for trend < 0.001). This decline was predominantly driven by younger women aged 18-44 years, in whom the prevalence fell from 27.2% of US adult women to 17.6% (*P* for trend < 0.001), whereas the decrease in men of the same age was marginal (14.9% of men in this age group to 12.6%; *P* for trend = 0.007). In addition, for the age group 45-64 years, the proportion of women in stages below 1 decreased from 10.6% to 6.6% of US women in this age group (*P* for trend < 0.001), with no significant change noted in men.

**Table 2:** Trends in Weighted Overall and Gender-/Age-stratified Prevalence of CKM Stages in US Adults, 1999-2020

	1999-2002 n = 194,019,985	2003-2008 n = 206,322,769	2009-2014 n = 221,705,110	2015-2020 n = 235,372,538	*P* for trend
**Weighted prevalence of overall US adults, % (95% CI)**					
**CKM S0+X**					
Women total	8.5 (7.1-9.9)	7.7 (6.7-8.7)	6.6 (5.8-7.4)	5.2 (4.1-6.2)	< 0.001
Men total	4.7 (3.8-5.6)	4.3 (3.7-5.0)	3.4 (2.8-4.0)	3.5 (2.7-4.3)	< 0.001
**CKM S1**					
Women total	7.0 (5.8-8.2)	11.6 (10.5-12.7)	10.8 (9.6-12.0)	10.6 (9.5-11.7)	0.29
Men total	9.8 (8.3-11.3)	13.4 (12.2-14.6)	11.2 (10.4-12.0)	11.0 (9.6-12.4)	0.93
**CKM S2**					
Women total	28.2 (26.6-29.8)	24.6 (13.3-25.9)	26.6 (25.3-27.9)	27.4 (26.1-28.7)	0.94
Men total	28.6 (27.0-30.2)	25.4 (24.1-26.7)	26.9 (25.6-28.2)	26.9 (25.2-28.6)	0.54
**CKM S3+4**					
Women total	7.3 (6.3-8.3)	7.2 (6.3-8.1)	7.5 (6.7-8.3)	8.0 (6.9-9.1)	0.034
Men total	5.9 (5.1-6.7)	5.6 (5.0-6.2)	7.1 (6.4-7.8)	7.4 (6.4-8.4)	0.003
**Weighted Prevalence of Women or Men in each Age Group, % (95% CI)**					
**CKM S0+X**					
Women 18-44	27.2 (22.8-31.6)	25.5 (22.3-28.7)	22.3 (19.7-25.1)	17.6 (14.6-20.6)	< 0.001
Men 18-44	14.9 (11.5-18.3)	14.7 (12.3-17.0)	12.2 (9.9-14.4)	12.6 (9.1-16.1)	0.007
Women 45-64	10.6 (7.6-13.6)	9.0 (6.8-11.1)	6.9 (4.7-9.1)	6.6 (3.6-9.5)	< 0.001
Men 45-64	4.2 (2.0-6.3)	4.2 (2.5-5.8)	2.9 (1.7-4.0)	3.5 (1.7-5.3)	0.16
Women ≥65	0.7 (0-1.4)^a^	2.0 (0.74-3.3)	2.0 (0.8-3.2)	1.7 (0.7-2.7)	0.25
Men ≥65	2.2 (0.8-3.5)	1.5 (0.3-2.7)	0.9 (0-2.2)^a^	0.6 (0-1.5)^a^	< 0.001
**CKM S1**					
Women 18-44	17.3 (13.9-20.7)	25.6 (22.8-28.4)	27.3 (24.5-30.1)	29.8 (26.4-3.2)	< 0.001
Men 18-44	24.6 (20.4-28.8)	32.8 (30.0-35.6)	32.0 (29.6-34.4)	32.4 (29.1-35.7)	0.15
Women 45-64	14.1 (10.2-18.0)	25.1 (21.3-28.9)	20.8 (16.9-24.7)	18.2 (15.0-21.4)	0.8
Men 45-64	16.1 (11.6-20.6)	26.8 (22.6-31.0)	18.5 (15.7-21.3)	17.4 (13.5-21.3)	0.81
Women ≥65	4.4 (2.0-6.8)	10.9 (7.7-14.1)	7.2 (5.3-9.1)	8.1 (5.1-11.1)	0.64
Men ≥65	11.0 (7.1-14.9)	11.5 (8.2-14.8)	6.6 (4.0-9.2)	8.3 (4.8-11.8)	0.11
**CKM S2**					
Women 18-44	40.0 (35.9-44.1)	36.9 (33.6-40.2)	37.4 (35.0-39.8)	38.8 (35.4-42.2)	0.59
Men 18-44	53.2 (49.8-56.6)	47.9 (44.9-50.9)	48.4 (45.1-51.7)	48.6 (44.1-53.1)	0.16
Women 45-64	66.1 (61.5-70.7)	54.9 (51.0-58.8)	60.3 (56.1-64.5)	63.2 (59.5-66.9)	0.89
Men 45-64	66.7 (60.5-72.9)	58.5 (54.3-62.7)	65.6 (61.8-69.4)	60.6 (54.8-66.4)	0.55
Women ≥65	73.9 (70.5-77.3)	62.1 (57.9-66.3)	67.9 (63.1-72.7)	64.8 (59.7-69.9)	0.35
Men ≥65	58.7 (51.6-65.8)	50.0 (45.4-54.6)	54.5 (50.5-58.5)	60.5 (54.5-66.5)	0.7
**CKM S3-4**					
Women 18-44	15.5 (13.2-17.8)	12.0 (9.5-14.5)	12.9 (10.8-15.0)	13.8 (11.8-15.8)	0.59
Men 18-44	7.3 (5.1-9.5)	4.7 (3.6-5.8)	7.4 (5.8-9.0)	6.4 (4.8-8.0)	0.99
Women 45-64	9.2 (6.0-12.4)	11.0 (8.8-13.2)	12.0 (9.7-14.3)	12.0 (9.5-14.5)	0.003
Men 45-64	13.0 (9.4-16.6)	10.5 (8.0-13.0)	13.1 (11.1-15.1)	18.5 (15.0-22.0)	0.11
Women ≥65	21.1 (18.0-24.2)	25.0 (20.2-29.8)	22.9 (18.6-27.2)	25.3 (20.0-30.6)	0.22
Men ≥65	28.1 (22.2-34.0)	37.0 (32.7-41.3)	38.0 (34.2-41.8)	30.5 (25.7-35.3)	0.81

*Note:* Upper part: percentages refer to all US adults of each period. The percentage of each column adds up to 100%. Lower part: percentages refer to the respective age and gender group across all stages for each period. The percentages of each of the 6 age/gender groups across all stages sum to 100%. Eg, in the 1999-2002 period, 27.2% of all women aged 18-44 were in CKMS stage 0+X, whereas in the 2015-2020 period, 17.6% of all women aged 18-45 were in stage 0+X.

Abbreviations: CKM, cardiovascular-kidney-metabolic syndrome; S0, stage 0 without any CKM risk factors; SX, stage X with risk (isolated abnormal HDL-cholesterol or LDL-cholesterol that is not defined in the CKM system); S1, stage 1 with excess or dysfunctional adiposity without subclinical or clinical CVD; S2, stage 2 with metabolic risk factors and/or moderate- to high-risk CKD without subclinical or clinical CVD; S3-4, stage 3 and 4 with subclinical or clinical CVD.

a Estimate may be unreliable with relative standard error >30%.

In younger women aged 18-44 years, the decrease in stages without CKM defining factors was accompanied by an increase in stage 1 prevalence, rising from 17.3% to 29.8% of US women in age group 18-44 years (*P* for trend < 0.001).

There was a noteworthy rise in the proportion of participants classified into CKM stage 3-4, increasing from 7.3% of the US adults to 8.0% (*P* for trend = 0.034) in women and from 5.9% to 7.4% in men (*P* for trend = 0.003). In particular, the confidence intervals for women and men aged 65 years and older were distinct in the periods 2003-2008 and 2009-2014 with higher prevalence (37% [95% CI, 32.7%-41.3%] and 38% [95% CI, 34.2%-41.8%]) in men than in women (25% [95% CI, 20.2%-29.8%] and 22.9 [95% CI, 18.6%-27.2%]), but they largely overlap in recent years (30.5 [95% CI, 25.7%-35.3%] in men and 25.3 [95% CI, 20%-30.6%] in women), suggesting a reduced advantage of elderly women.

### Prevalence of Metabolic Risk Factors and CKD in Stage 2 by Gender

We specifically explored stage 2, as it reflects the highest likelihood of progression to subclinical or clinical CVD and comprises more than half of the population.

Among all US adults with CKM stage 2, a higher proportion of women experienced CKD with moderate to high risk compared with men (9.8% of US adults in stage 2 vs 6.6%; *P* = 0.006). This trend was consistent across all age groups, with a particularly notable difference in women aged 65 years and older (23.6% of US adults in stage 2 with CKD vs 13.9% in men; *P* < 0.001) (Figs S1 and S2). Overall, hypertriglyceridemia was less common in women (*P* = 0.025). However, accounting for age, a significantly higher proportion of women over 65 years were affected (11.0% of US adults in stage 2 with hypertriglyceridemia vs 5.9% in men, *P* < 0.001) (Figs S1 and S3). Younger women had lower hypertension rates than men (11.2% of US adults in stage 2 with hypertension vs 18.8%; *P* < 0.001). In contrast, among those aged 65 years and older, hypertension was more common in women (15.2% vs 9.9% of US adults in stage 2 with hypertension; *P* < 0.001) (Fig S4). Women over 65 years carried a higher burden of metabolic syndrome (MetS) (11.3% of US adults in stage 2 with MetS vs 6.6% in men; *P* < 0.001) (Fig S5). The prevalence of diabetes was similar among genders in all age groups (Fig S6).

### Mortality According to Age, Gender, and CKM Syndrome Stages

To examine potential age- and sex-related differences in how CKM stage translates into long-term outcomes, we analyzed all-cause and cardiovascular mortality stratified by CKM stage, age, and sex (Tables S3 and S4). Across all age groups and CKM stages, women exhibited a higher hazard associated with advancing CKM stage compared with stage 0. This trend was observed consistently across CKM syndrome stages and age groups and reached statistical significance under certain conditions.

### Sensitivity Analysis

After reweighting for nonresponse, we observed no significant deviation from our primary analysis (Tables S5 and S6). The aggregated outcome from 5 imputations, following the imputation of missing data, aligned consistently with the primary analysis (Tables S5 and S7).

## Discussion

Our analysis of this nationally representative survey of US adults reveals gender-related and age-related disparities in the prevalence of different CKM syndrome stages in the general population that changed over the last 20 years. Women under 45 years showed a higher prevalence of being in stages confining no risk compared with men but also showed a higher prevalence in stages with already established targeted organ damage. From 1999 to March 2020, the prevalence of CKM stages with low risk (below stage 1) decreased, with younger women experiencing a more pronounced decline when compared with men. In addition, elderly women lost most of their advantage as the prevalence of stages 3 and 4 neared the prevalence in men of this age.

Several studies have used NHANES to characterize the CKM syndrome in the US population.^4^^,^^20^, ^21^, ^22^ However, most did not analyze gender-age interaction or, if they did, they did not examine how these interactions evolve over time. In addition, these studies varied in the periods analyzed, ranging from 1988-2018^22^ to 2011-2020.^4^ Given the importance of both the temporal trends and gender-age interactions, direct comparisons between these studies and our findings are difficult. Indeed, many of the differences may stem from variations in the time periods analyzed, which can introduce methodological inconsistences, particularly using very early NHANES data, such as differences in laboratory measurements, or from inclusion criteria, such as age restrictions. Our study not only provides the longest but also the most methodologically appropriate time course for analysis and is the only one to assess temporal changes in relation to gender-age interaction.

The CKM syndrome is an evolving condition that begins already in childhood.^23^^,^^24^ Current evidence demonstrates that trends in CKM syndrome have been increasing in young adulthood despite the general improvement of health care.^1^^,^^25^, ^26^, ^27^ It is generally believed that younger women exhibit lower cardiovascular risk when compared with men,^5^ which is reflected by the higher proportion of women without CKM defining factors. However, our analysis also reveals that more young women are at an elevated risk for target organ damage (stage 3-4), compared with their male counterparts. This result may seem surprising; nevertheless, several observations may help to elucidate this finding. We have shown that healthy children and adolescent women are more susceptible to develop left ventricular hypertrophy with increasing BMI than men,^24^ and that girls with CKD are more likely to develop increased arterial stiffness than boys.^28^ Among other observations, Ji et al^29^ showed that women in their thirties are more likely to develop a more rapid increase in blood pressure than men. These data underscore the urgent need to focus on CKM protection for younger women. When risk factors are present, women are more prone to developing target organ damage, even before menopause. Moreover, our analysis indicates that the protective advantage of younger women regarding fewer risk factors has diminished over the past 20 years. These observations imply an escalating burden of CKM syndrome as these younger women age, unless current adverse trends in CKM risk factors can be reversed.

To reinforce initiatives promoting the CKM health of young adults of all genders, health care providers could collaborate with patients to provide personalized medical advice by integrating digital health data,^30^^,^^31^ since young adults may be more motivated to access their health records using mobile and wearable devices. Young adulthood is a critical period for the establishment of lifestyle behaviors that have long-term implications for cardiovascular health. Changes during this period are associated with subclinical atherosclerosis risk in middle age—healthy changes reduce the risk, whereas unhealthy changes increase it.^32^ Thus, education on healthy lifestyles and the promotion of lifestyle modifications are essential for preventing the elements of the CKM syndrome. In addition, more CKM health trials should focus on including participants under 45 years, especially women, as young women have been underrepresented in behavioral and pharmacologic prevention/intervention trials compared with young men.^33^, ^34^, ^35^, ^36^, ^37^

For the effective management of CKM syndrome, it is essential to also consider gender and age disparities to prevent a progression into stage 3-4. In CKM syndrome stage 2, for example, as women reached an older age, they showed a significantly higher prevalence across all metabolic risk factors and CKD compared with men. Importantly, this higher prevalence persisted in CKD, regardless of age. According to the US Renal Data System 2023 Annual Report, women have experienced CKD more often than men since 2005-2008.^38^ Several studies demonstrated a significant deterioration in lipid profiles and an increasing prevalence of MetS with menopause, beyond the effects of chronological aging, leading to a remarkable CKM syndrome progression and thus an increase of CVD absolute risk after menopause.^39^, ^40^, ^41^ To address this unmet need referred to gender-disparities and age-disparities, additional efforts should be directed toward research fields, clinical practice, and guideline development.

In participants aged 45 years and older, ∼two-thirds were in stage 2, reflecting a substantial risk for CKM damage. Elderly women are notably overrepresented in this group and may be underrecognized.^42^ Moreover, there is also an obvious trend showing reduced benefits for women compared with men in prevalence of CVD. In our long-term outcome analysis, women also exhibited a greater excess mortality risk than men, with higher hazards for both all-cause and cardiovascular mortality across CKM syndrome stages 2-4 and age groups, findings consistent with those reported by Ji et al.^22^ This likely reflects that recent advancements in preventive treatments have been more effectively implemented in men, highlighting the ongoing or obviously even increasing gender disparity in cardiovascular management.^5^ Although there are significant challenges in managing poor CKM health, a comprehensive classification and risk assessment of CKM syndrome also provides us with opportunities to detect earlier stages for preventive measures and implement effective interventions, thereby decelerating progression. In addition, optimizing CKM health necessitates the integration of social determinants, behavioral interventions, early-life prevention, multidisciplinary care, and ensuring affordable access to pharmacotherapy.^43^, ^44^, ^45^

This study has several limitations. First, it comprises only the noninstitutionalized, civilian population and does not capture the individuals in nursing homes or the military. Second, we did not distinguish between diabetes mellitus types. This decision was based on our opinion that type 1 diabetes should also be included in CKM syndrome because of its association with CKD progression and higher cardiovascular risk. Third, the NHANES gender variable is diffuse, not distinguishing sex at birth, perceived gender, self-identification, or transgender status. Individuals outside binary categories were therefore not properly captured. However, since only a small proportion of the US population identifies as diverse or transgender, this is unlikely to affect our results. Furthermore, because of the absence of specific laboratory or interventional diagnostic data in NHANES, we cannot precisely define CKM syndrome stages 3 and 4 but rely on information from interviews. Finally, nonresponse could have influenced our conclusion. However, sensitivity analyses, one after reweighting for nonresponse and another after imputing missing data, demonstrated comparable results, which confirmed our results.

In conclusion, our results highlight gender-related and age-related differences in the prevalence and trends of CKM syndrome over the past 2 decades showing numerous disadvantages of women. This applies to elderly women, who now exhibit a similar prevalence of advanced CKM syndrome stages as men, as well as to younger women, who bear an even higher burden of progressed CKM syndrome and are increasingly less free of CKM risk factors over time. The key tasks now are to identify the biological, psychological, and socioeconomic reasons leading to this development and to transform these important findings into targeted preventive measures enforcing lifestyle measures und medical treatments that consider young and elderly women as the vulnerable groups.
