# Supplementary material for: Gender-related and Age-related Disparities in Prevalence of the Cardiovascular-Kidney-Metabolic Syndrome Among US Adults From 1999-2020: An Analysis of the NHANES Survey
Source: Kidney Med. 2025 Dec 26;8(3):101234. doi: 10.1016/j.xkme.2025.101234 (PMC12874573; doi:10.1016/j.xkme.2025.101234)
Supplement: Supplementary File (PDF) — Figures S1-S6; Tables S1-S7. [file mmc1.docx]

**Supplemental Material**

**Table S1** KDIGO Classification of CKD

**Table S2** Definition of CKM Stages 0-4

**Figure S1** Gender-related Prevalence of CKD, Diabetes, Hypertension, Hypertriglyceridemia and MetS in US Adults with CKM syndrome Stage 2

**Figure S2** Gender- and Age-related Prevalence of CKD within CKM Stage 2

**Figure S3** Gender- and Age-related Prevalence of Hypertriglyceridemia within CKM Stage 2

**Figure S4** Gender- and Age-related Prevalence of Hypertension within CKM Stage 2

**Figure S5** Gender- and Age-related Prevalence of MetS within CKM Stage 2

**Figure S6** Gender- and Age-related Prevalence of Diabetes within CKM Stage 2

**Table S3** 15-year Cumulative Incidence of All-cause and Cardiovascular Mortality According to Age, Gender and CKM Stages

**Table S4** Gender- and Age-stratified Association between All-Cause and Cardiovascular Mortality and CKM Syndrome Stages.

**Table S5** Weighted Population Characteristics Overall and by CKM Syndrome Stages in US Adults

**Table S6** Sensitivity Analysis with Reweighting for nonresponse

**Table S7** Sensitivity Analysis with multivariate multiple Imputation

|  | | | **Albuminuria categories** | | |  |
| --- | --- | --- | --- | --- | --- | --- |
|  |  |  | **A1** | **A2** | **A3** |  |
| **eGFR categories** | **Stage** | **eGFR, ml/min/1.73m^2^** | < 30 mg/g | 30-299 mg/g | ≥300 mg/g |  |
|  |  |  |  |  |  |  |
|  | **G1** | >=90 |  |  |  |  |
|  | **G2** | 60-89 |  |  |  |  |
|  | **G3a** | 45-59 |  |  |  |  |
|  | **G3b** | 30-44 |  |  |  |  |
|  | **G4** | 15-29 |  |  |  |  |
|  | **G5** | <15 |  |  |  |  |

|  | Low risk (if no other markers of kidney disease, no CKD) |  |  | | High Risk |
| --- | --- | --- | --- | --- | --- |
|  |  |  |  |  |  |
|  | Moderately increased risk |  |  |  | Very high risk |

**Table S1. KDIGO Classification of CKD (modified in accordance to KDIGO 2024 Guidelines)**

Abbreviations: eGFR, estimated glomerular filtration rate; CKD, chronic kidney disease.

| **CKM Syndrome Stages** | **Definition** |
| --- | --- |
| CKM Stage 0 | normal BMI 18.5 – 24,9 kg/m^2^ (17.5 – 22.9 kg/m^2^ if Asian ancestry); normal waist circumference < 88/102 cm in women/men or < 80/90 cm in women/men if Asian ancestry; normoglycemia with fasting blood glucose < 100 mg/dl or HbA1c < 5.7% without any glucose-lowering medication; normotension with systolic blood pressure < 130 mmHg or diastolic blood pressure < 80 mmHg without any antihypertensive medication; a normal lipid profile with HDL cholesterol ≥ 40 mg/dl for men and ≥ 50 mg/dl for women, LDL cholesterol < 130 mg/dl, triglycerides < 150 mg/dl without any lipid-lowering medication; no CKD according to KDIGO 2024 Guidelines and no subclinical or clinical CVD according to self-report |
| CKM stage X | Individuals with LDL cholesterol ≥130 mg/dl and/or HDL cholesterol <40 mg/dl in men and <50 mg/dl in women, but no additional risk factors placing them into stages 1-4 |
| CKM Stage 1 | BMI ≥ 25 kg/m^2^ or ≥ 23 kg/m^2^ if Asian ancestry; waist circumference ≥ 88/102 cm in women/men or ≥ 80/90 cm in women/men if Asian ancestry; fasting blood glucose ≥ 100-124 mg/dl or HbA1c 5.7%-6.4%; no CKD according to KDIGO 2024 Guideline and no subclinical or clinical CVD according to self-report. |
| CKM Stage 2 | Individuals with metabolic risk factors: hypertriglyceridemia ≥ 135 mg/dl; hypertension with systolic blood pressure ≥ 130 mmHg or diastolic blood pressure ≥ 80 mmHg and/or with any antihypertensive medication; MetS (presence of 3 or more of the following: 1) waist circumference ≥ 88/102 cm in women/men or ≥ 80/90 cm in women/men if Asian ancestry; 2) HDL cholesterol < 40 mg/dl for men and < 50 mg/dl for women; 3) triglycerides ≥ 150 mg/dl; 4) systolic blood pressure ≥ 130 mmHg or diastolic blood pressure ≥ 80 mmHg and/or use of antihypertensive medications; 5) fasting blood glucose ≥ 100 mg/dl; Diabetes with HbA1c ≥ 6.5% or fasting blood glucose ≥ 126 mg/dl and/or with any glucose-lowering medication; or individuals with moderate- to high-risk CKD according to KDIGO 2024 Guidelines;  no subclinical or clinical CVD according to self-report. |
| CKM Stage 3 | Subclinical ASCVD or subclinical HF among individuals with excess/dysfunctional adiposity, other metabolic risk factors, or CKD with very high-risk according to KDIGO 2024 Guidelines. |
| CKM Stage 4 | Clinical CVD among individuals with excess/dysfunctional adiposity, other CKM risk factors, or CKD according to KDIGO 2024 Guidelines. |

**Table S2. Definition of CKM Stages 0-4 (modified in accordance to Ndumele et. al.)**

Abbreviations: ASCVD, atherosclerotic cardiovascular disease; BMI, body mass index; CKM, cardiovascular-kidney-metabolic syndrome; CKD, chronic kidney disease; CVD, cardiovascular disease; HbA1c, hemoglobin A1c; HDL, high-density lipoprotein; HF, heart failure; MetS, metabolic syndrome.

**Figure S1**. **Gender-related Prevalence of CKD, Diabetes, Hypertension, Hypertriglyceridemia and MetS in US Adults with CKM syndrome Stage 2**

Prevalence estimates are presented as weighted prevalence of noninstitutionalized US adults in stage 2.

CKM, cardiovascular-kidney-metabolic syndrome; CKD, chronic kidney disease with moderate and high risk; Abbreviations: MetS, metabolic syndrome.
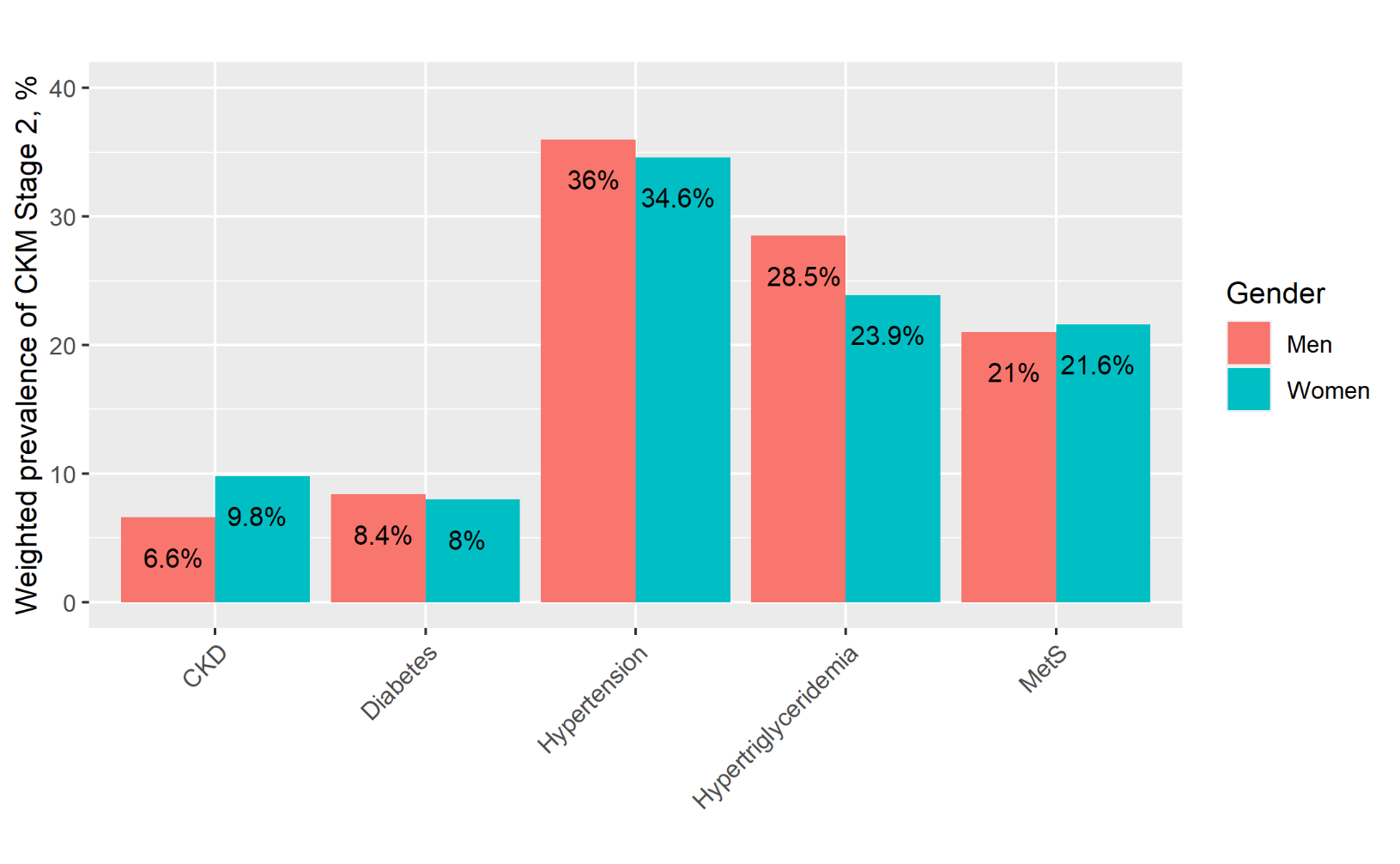


**
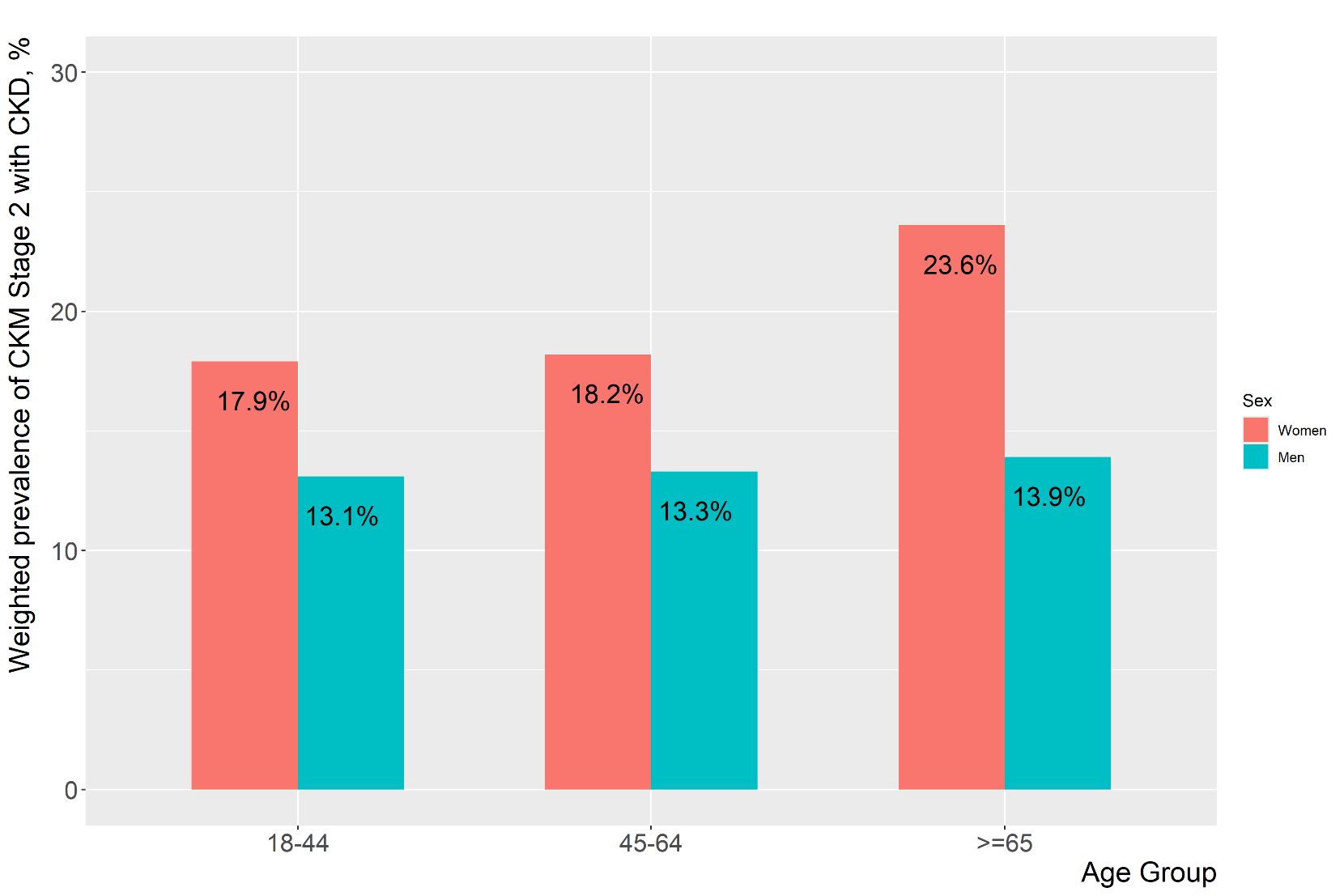
Figure S2. Gender- and Age-related Prevalence of CKD within CKM Stage 2**

Prevalence of women and men between 18-44 years: 17.9% and 13.1%, P < 0.001; prevalence of women and men between 45-64 years: 18.2% and 13.3%, P < 0.001; prevalence of women and men aged 65 and older: 23.6% and 13.9%, P < 0.001. All prevalence estimates are presented as weighted prevalence of noninstitutionalized US adults in stage 2 with CKD.

Abbreviations: CKD, chronic kidney disease with moderate to high risk; CKM, cardiovascular-kidney-metabolic syndrome.

**
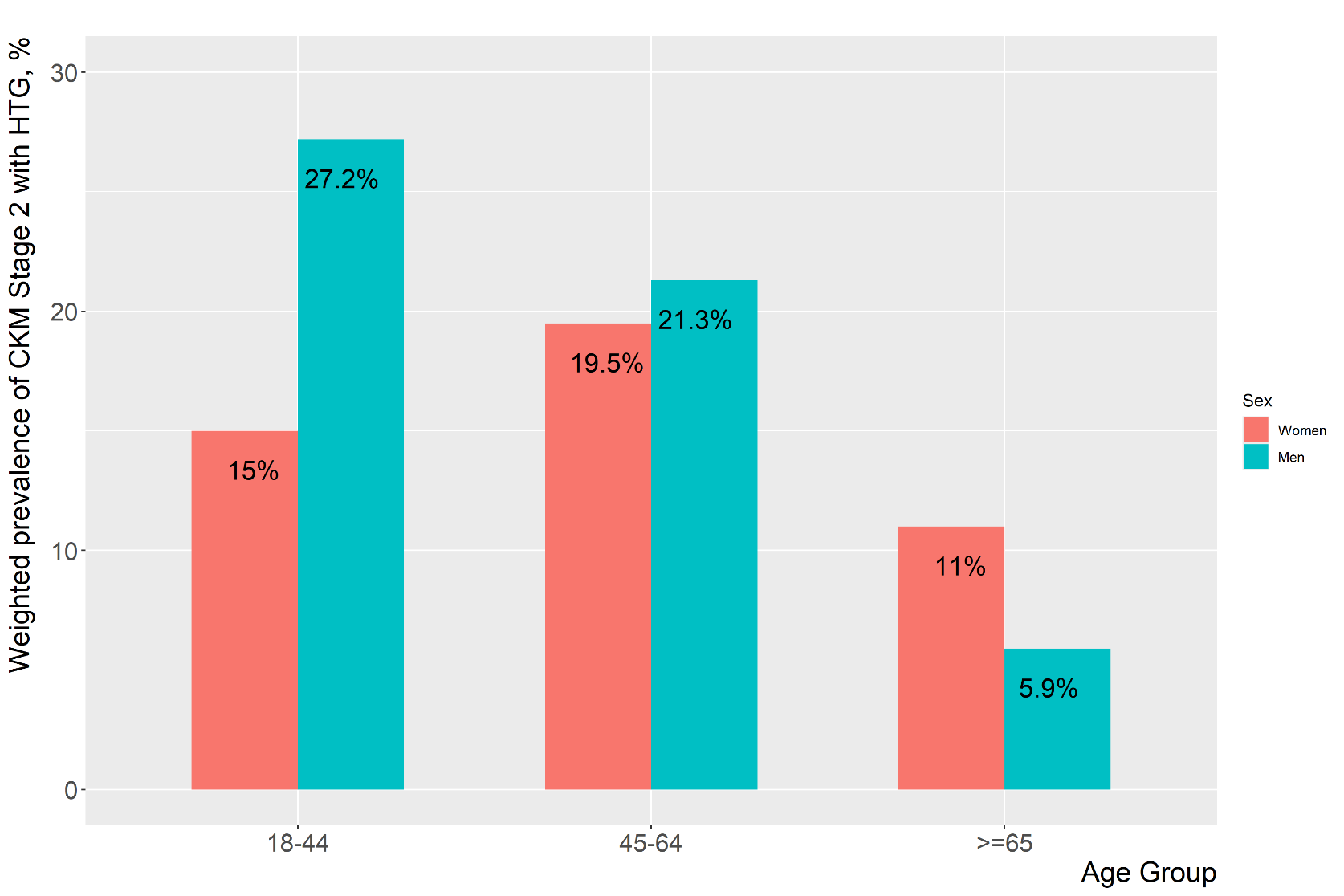
Figure S3.** **Gender- and Age- related Prevalence of Hypertriglyceridemia within CKM Stage 2**

Prevalence of women and men between 18-24 years: 15.0% and 27.2% (*P* < 0.001); prevalence of women and men between 45-64 years: 19.5% and 21.3% (*P* = 0.06); prevalence of women and men aged 65 or older: 11.0% and 5.9% (*P* < 0.001). All prevalence estimates are presented as weighted prevalence of noninstitutionalized US adults in stage 2 with hypertriglyceridemia.

Abbreviations: HTG, hypertriglyceridemia; CKM, cardiovascular-kidney-metabolic syndrome.


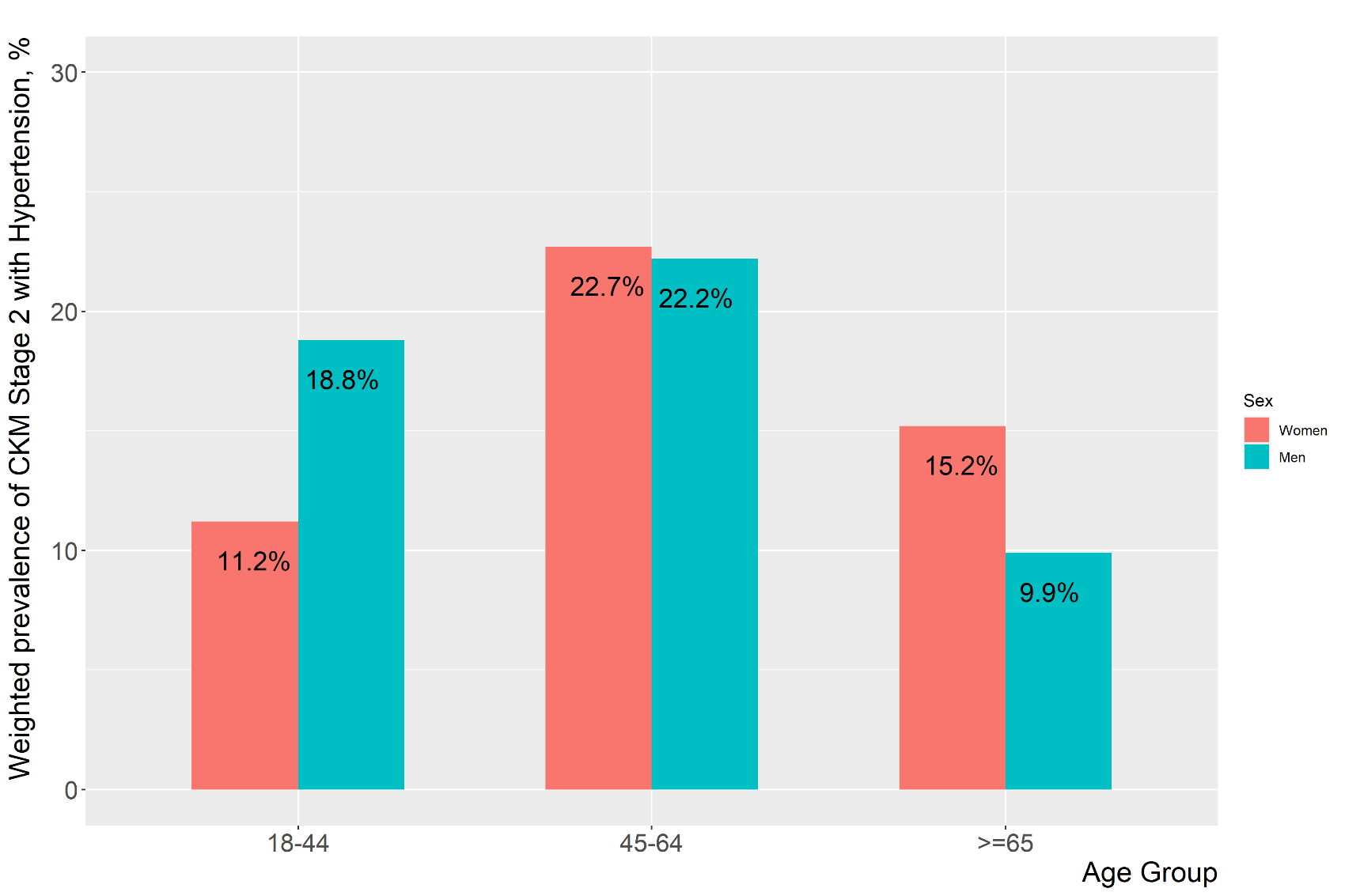
**Figure S4. Gender- and Age-related Prevalence of Hypertension within CKM Stage 2**

Prevalence of women and men between 18-44 years: 11.2% and 18.8% (*P* < 0.001); prevalence of women and men between 45-64 years: 22.7% and 22.2% (*P* = 0.578); prevalence of women and men aged 65 or older: 15.2% and 9.9% (*P* < 0.001). All prevalence estimates are presented as weighted prevalence of noninstitutionalized US adults in stage 2 with hypertension.

Abbreviations: CKM, cardiovascular-kidney-metabolic syndrome.


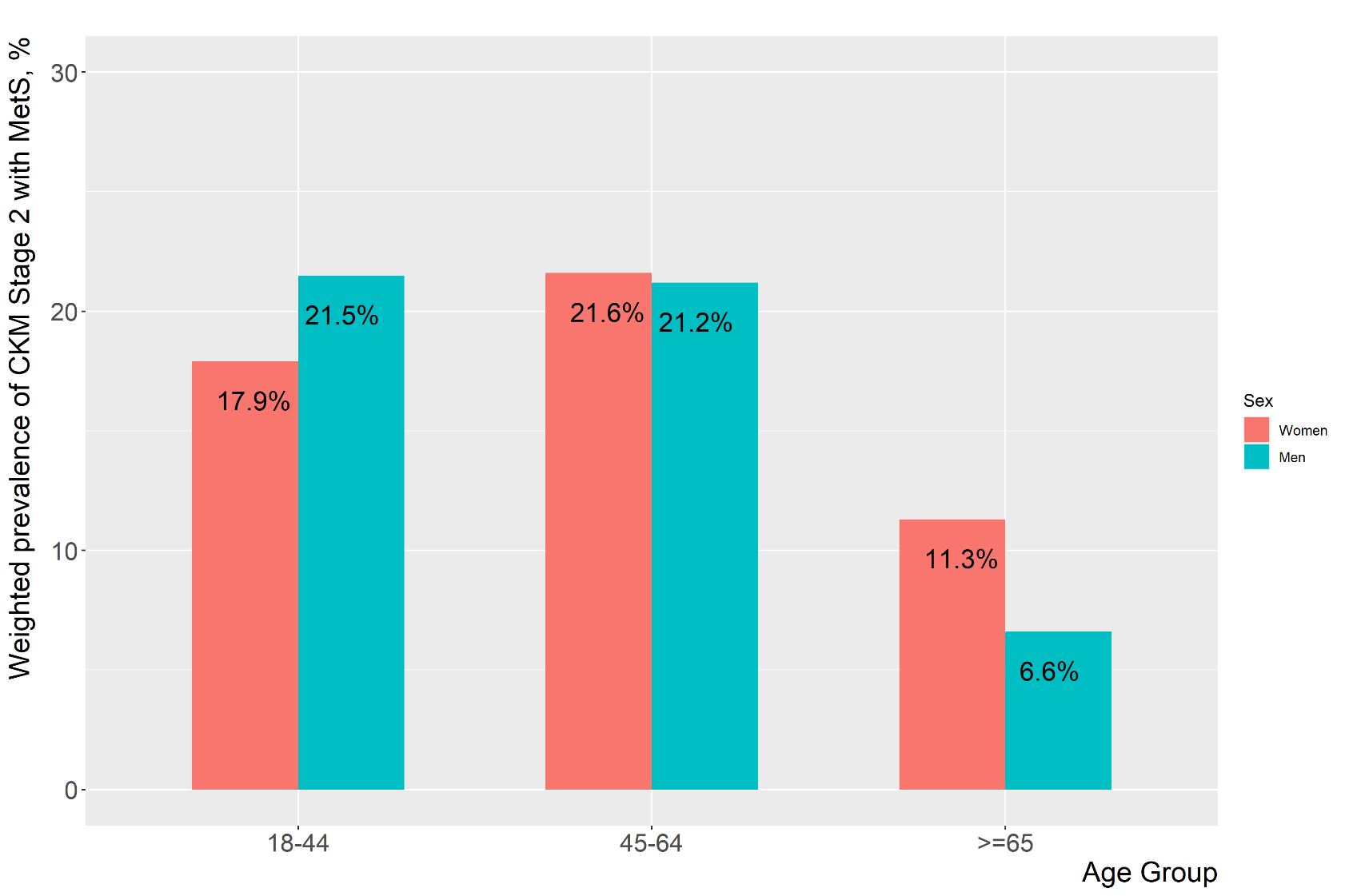
**Figure S5. Gender- and Age-related Prevalence of MetS within CKM Stage 2**

Prevalence of women and men between 18-44 years: 17.9% and 21.5% (*P* = 0.0003); prevalence of women and men between 45-64 years: 21.6% and 21.2% (*P* = 0.713); prevalence of women and men aged 65 or older: 11.3% and 6.6% (*P* < 0.001). All prevalence estimates are presented as weighted prevalence of noninstitutionalized US adults in stage 2 with metabolic syndrome.

Abbreviations: MetS, metabolic syndrome.


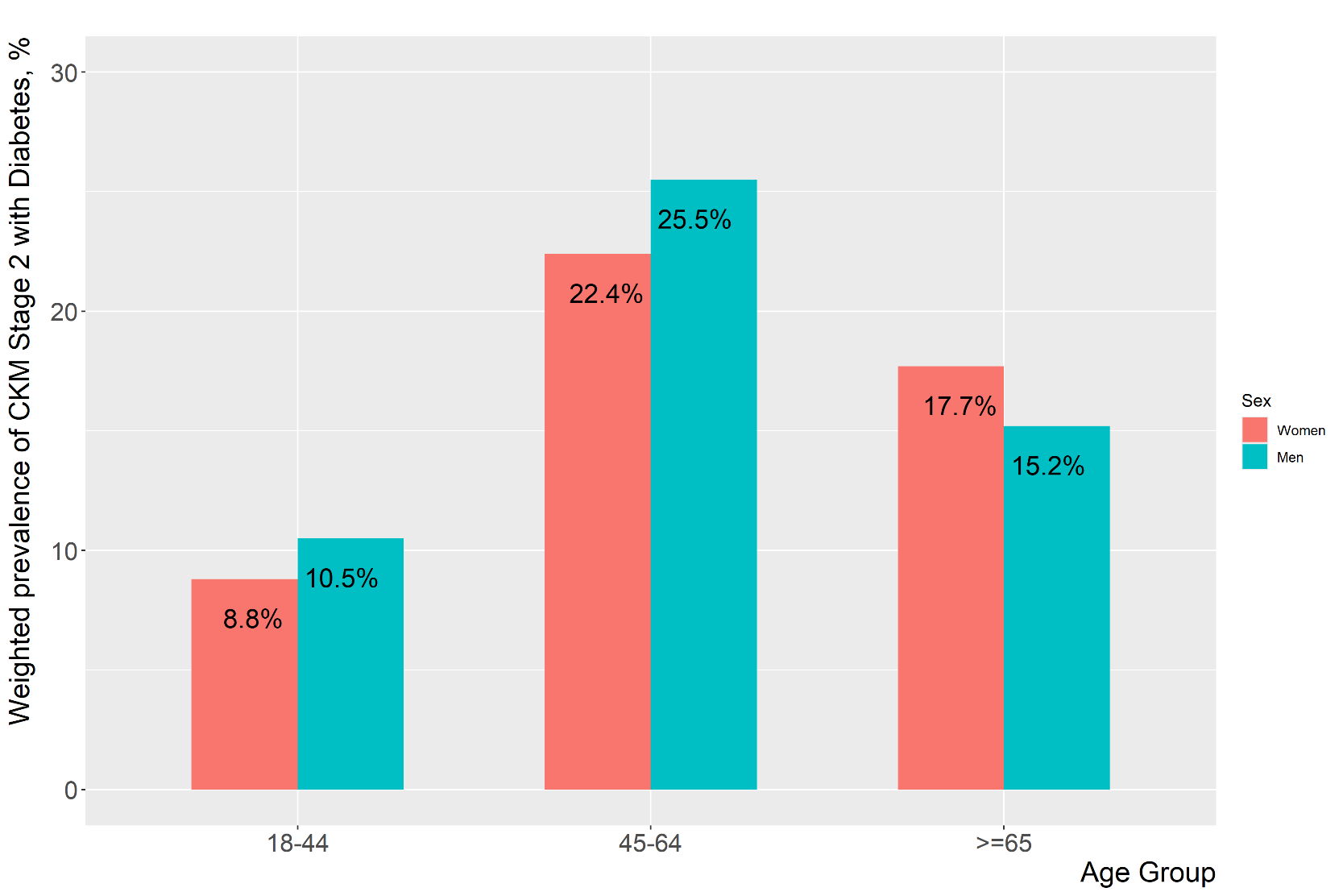
**Figure S6. Gender- and Age-related Prevalence of Diabetes within CKM Stage 2**

Prevalence of women and men between 18-44 years: 8.8% and 10.5% (*P* = 0.134); prevalence of women and men between 45-64 years: 22.4% and 25.5% (*P* = 0.063); prevalence of women and men aged 65 or older: 17.7% and 15.2% (*P* = 0.079). All prevalence estimates are presented as weighted prevalence of noninstitutionalized US adults in stage 2 with diabetes.

Abbreviations: CKM, cardiovascular-kidney-metabolic syndrome.

| **Age Group** | **Sex** | **15-year Cumulative Incidences of All-Cause Mortality and Cardiovascular Death** | | | | | | | |
| --- | --- | --- | --- | --- | --- | --- | --- | --- | --- |
|  |  | **CKM Syndrome Stage 0** | | **CKM Syndrome Stage 1** | | **CKM Syndrome Stage 2** | | **CKM Syndrome Stage 3/4** | |
|  |  | **ACM** | **CV Death** | **ACM** | **CV Death** | **ACM** | **CV Death** | **ACM** | **CV Death** |
| **Overall** | Women | 1.4% (0.4 - 2.4) | 0.6% (0* - 1.4) | 3.9% (2.1 - 5.8) | 2.2% (0.8 - 3.6) | 12.1% (10.7 - 13.4) | 6.0% (5.1 - 6.9) | 23.1 (17.4 - 28.8) | 28.5% (25.8 - 31.3) |
|  | Men | 3.9% (2.2 - 5.5) | 3.1% (1.5 - 4.7) | 5.8% (4.0 - 7.6) | 2.8% (1.8 - 3.8) | 11.4% (10.0 - 12.8) | 5.7% (4.7 - 6.7) | 40.7% (34.8 - 46.6) | 35.9% (32.9 - 39.0) |
| **Age 18 - 44** | Women | 0.6% (0* - 1.2) | 0.8% (0* - 2.4) | 1.4% (0.2 - 2.7) | 0.5% (0* - 1.1) | 3.5% (1.9 - 5.1) | 1.6% (0.6 - 2.5) | 4.7% (2.1 - 7.3) | 2.6% (0.5 - 4.7) |
|  | Men | 2.8% (0.7 - 4.9) | 2.4% (0.6 - 4.2) | 3.3% (1.7 - 4.9) | 1.8% (0.7 - 2.9) | 3.6% (2.4 - 4.8) | 1.9% (1.1 - 2.8) | 8.0% (2.4 - 13.6) | 3.7% (0.7 - 6.6) |
| **Age 45 - 64** | Women | 3.2% (0* - 6.9) | 1.7% (0* - 5.0) | 4.3% (1.1 - 7.5) | 2.9% (0*- 5.9) | 8.4% (6.5 - 10.2) | 3.7% (2.4 - 5.0) | 23.1% (17.4 - 28.8) | 16.1% (11.1 - 21.2) |
|  | Men | 4.7% (0.1 - 9.4) | 3.4% (0* - 7.8) | 7.2% (2.4 - 12.0) | 2.6% (0.5 - 4.7) | 14.5% (12.0 - 17.1) | 6.8% (5.1 - 8.6) | 40.7% (34.8 - 46.6) | 25.2% (18.7 - 31.8) |
| **Age >=65** | Women | 23.7% (5.6 - 41.8) | 13.5% (0* - 27.8) | 41.5% (20.4 - 62.6) | 25.2% (5.6 - 44.8) | 39.3% (34.7 - 44.0) | 23.0% (18.7 - 27.4) | 74.3% (70.8 - 77.8) | 58.1% (53.7 - 62.6) |
|  | Men | 57.9% (25.7 - 90.2) | 50.3% (14.3 - 86.2) | 32.4% (18.9 - 45.9) | 18.7 (6.6 - 30.9) | 43.3% (36.9 - 49.7) | 25.9% (19.8 - 32.0) | 77.0% (74.1 - 79.8) | 59.2% (55.1- 63.2) |

**Table S3.** **15-year Cumulative Incidence of All-cause and Cardiovascular Mortality According to Age, Gender and CKM Stages**

*Estimate may be unreliable with relative standard error >30%

Abbreviations: ACM, All-cause mortality; CKMS, cardiovascular-kidney-metabolic syndrome; CV, cardiovascular.

| **Age Group** | **Sex** | **Cox Proportional Hazards Model Crude, HR (95% CI)** | | | | | | | | |
| --- | --- | --- | --- | --- | --- | --- | --- | --- | --- | --- |
|  |  | **CKM Syndrome Stage 0** | | **CKM Syndrome Stage 1** | | **CKM Syndrome Stage 2** | | | **CKM Syndrome Stage 3/4** | |
|  |  | **ACM** | **CV Death** | **ACM** | **CV Death** | **ACM** | | **CV Death** | **ACM** | **CV Death** |
| **Overall** | Women | referent | | 2.20 (1.13 - 4.28) | 2.35 (0.86 - 6.40) | 7.65 (4.39 - 13.33) | 7.62 (3.15 - 18.43) | | 32.30 (18.45 - 56.53) | 43.33 (18.05 - 104.02) |
|  | Men | referent | | 1.52 (0.87 - 2.64) | 1.05 (0.53 - 2.07) | 3.27 (2.14 -4.99) | 2.36 (1.42 - 3.92) | | 19.90 (12.93 - 30.65) | 16.32 (9.79 - 27.23) |
|  | *P* interaction | referent | | 0.355 | 0.198 | 0.008* | 0.024* | | 0.15 | 0.063 |
| **Age 18 - 44** | Women | referent | | 1.50 (0.43 - 5.28) | 1.52 (0.27 - 8.7) | 3.98 (1.49 - 10.62) | 3.16 (0.81 - 12.27) | | 5.23 (1.87 - 14.64) | 4.91 (0.99 - 24.27) |
|  | Men | referent | | 1.25 (0.55 - 2.82) | 0.86 (0.34 - 2.20) | 1.48 (0.70 - 3.17) | 1.06 (0.49 - 2.29) | | 2.20 (0.84 - 5.81) | 1.56 (0.53 - 4.65) |
|  | *P* interaction | referent | | 0.798 | 0.545 | 0.053 | 0.109 | | 0.188 | 0.202 |
| **Age 45 - 64** | Women | referent | | 1.99 (0.74 - 5.33) | 2.83 (0.35 - 22.7) | 3.56 (1.40 - 9.05) | 4.75 (0.71 - 31.67) | | 11.66 (4.59 - 29.61) | 21.89 (3.31 - 144.59) |
|  | Men | referent | | 1.40 (0.62 - 3.18) | 0.98 (0.25 - 3.82) | 3.40(1.40 - 8.23) | 2.68 (0.77 - 9.36) | | 10.66 (4.51 - 25.22) | 9.33 (2.71 - 32.20) |
|  | *P* interaction | referent | | 0.599 | 0.404 | 0.941 | 0.627 | | 0.901 | 0.459 |
| **Age >=65** | Women | referent | | 1.17 (0.38 - 3.58) | 1.18 (0.27 - 5.06) | 1.72 (0.65 - 4.57) | 1.58 (0.45 - 5.59) | | 5.23 (1.96 - 13.90) | 6.18 (1.79 - 21.38) |
|  | Men | referent | | 0.62 (0.20 - 1.91) | 0.52 (0.13 - 2.07) | 0.91 (0.34 - 2.48) | 0.83 (0.23 - 2.94) | | 2.54 (0.93 - 6.92) | 2.57 (0.73 - 9.00) |
|  | *P* interaction | referent | | 0.402 | 0.417 | 0.352 | 0.47 | | 0.309 | 0.328 |
| **Age Group** | **Sex** | **Cox Proportional Hazards Model Adjusted for Sociodemographic Factors, Smoking Status and Cycle Year, aHR (95% CI)** | | | | | | | | |
|  |  | **CKM Syndrome Stage 0** | | **CKM Syndrome Stage 1** | | **CKM Syndrome Stage 2** | | | **CKM Syndrome Stage 3/4** | |
|  |  | **ACM** | **CV Death** | **ACM** | **CV Death** | **ACM** | **CV Death** | | **ACM** | **CV Death** |
| **Overall** | Women | referent | | 1.50 (0.77 - 2.90) | 1.56 (0.57 - 4.24) | 2.36 (1.37 - 4.06) | 2.16 (0.90 - 5.17) | | 4.92 (2.82 - 8.61) | 5.83 (2.42 - 14.01) |
|  | Men | referent | | 0.98 (0.56 - 1.72) | 0.67 (0.34 - 1.33) | 1.50 (0.95 - 2.26) | 1.08 (0.64 - 1.82) | | 3.07 (1.90 - 4.96) | 2.53 (1.48 - 4.33) |
|  | *P* interaction | referent | | 0.186 | 0.122 | 0.053 | 0.067 | | 0.023* | 0.014* |
| **Age 18 - 44** | Women | referent | | 1.48 (0.43 - 5.16) | 1.35 (0.24 - 7.50) | 3.32 (1.20 - 9.19) | 2.42 (0.60 - 9.71) | | 4.95 (1.80 - 13.62) | 4.57 (0.95 - 22.00) |
|  | Men | referent | | 1.23 (0.55 - 2.73) | 0.81 (0.33 - 2.02) | 1.28 (0.61 - 2.69) | 0.95 (0.45 - 3.85) | | 1.86 (0.72 - 4.83) | 1.35 (0.47 - 3.85) |
|  | *P* interaction | referent | | 0.745 | 0.523 | 0.043 | 0.104 | | 0.132 | 0.159 |
| **Age 45 - 64** | Women | referent | | 1.80 (0.68 - 4.77) | 2.61 (0.32 - 20.86) | 2.70 (1.06 - 6.85) | 3.71 (0.55 - 24.87) | | 8.03 (3.12 - 20.64) | 15.92 (2.39 - 106.25) |
|  | Men | referent | | 1.37 (0.60 - 3.11) | 0.99 (0.25 - 3.91) | 2.97 (1.24 - 7.12) | 2.47 (0.70 - 8.64) | | 7.26 (3.04 - 17.36) | 6.92 (1.97 - 24.26) |
|  | *P* interaction | referent | | 0.667 | 0.433 | 0.891 | 0.704 | | 0.864 | 0.454 |
| **Age >=65** | Women | referent | | 1.13 (0.39 - 3.34) | 1.13 (0.28 - 4.67) | 1.55 (0.61 - 3.90) | 1.38 (0.41 - 4.67) | | 2.71 (1.07 - 6.89) | 2.99 (0.90 - 9.95) |
|  | Men | referent | | 0.61 (0.19 - 1.99) | 0.53 (0.13 - 2.19) | 1.07 (0.38 - 3.06) | 1.01 (0.26 - 3.84) | | 1.73 (0.59 - 5.06) | 1.74 (0.45 - 6.72) |
|  | *P* interaction | referent | | 0.429 | 0.451 | 0.631 | 0.738 | | 0.532 | 0.519 |

**Table S4.** **Gender- and Age-stratified Association between All-Cause and Cardiovascular Mortality and CKM Syndrome Stages.**

Abbreviations: ACM, All-cause mortality; CKMS, cardiovascular-kidney-metabolic syndrome; CV, cardiovascular

|  | **Overall** | | | **CKM Stage 0** | **CKM Stage 1** | **CKM Stage 2** |
| --- | --- | --- | --- | --- | --- | --- |
| **Unweighted Nr.** | 32848 | | | 3487 | 5703 | 17757 |
| **Weighted Population** | 215480397 | | | 17128893 | 39464629 | 121882777 |
| **Variable** | **Weighted Prevalence % (95% CI)** | | | | | |
| **Overall** | 100 |  |  | 7.9 (7.4-8.5) | 18.3 (17.6-19.1) | 56.6 (55.6-57.5) |
| **Women** | 51.3 (50.6-51.9) | | | 64.0 (61.1-66.9) | 48.8 (46.7-50.8) | 49.3 (48.3-50.2) |
| **Men** | 48.7 (48.1-49.4) | | | 36.0 (33.1-38.9) | 51.2 (49.2-53.3) | 50.7 (49.8-51.7) |
| **Age 18-24** | 9.2 (8.6-9.8) | | | 23.0 (20.6-25.5) | 13.2 (11.8-14.5) | 5.7 (5.1-6.4) |
| **Age 25-44** | 37.1 (36.1-38.2) | | | 57.8 (54.3-61.3) | 50.2 (47.9-52.5) | 32.8 (31.5-34.0) |
| **Age 45-64** | 35.6 (34.6-36.5) | | | 17.0 (14.4-19.6) | 30.6 (28.4-32.7) | 41.1 (39.9-42.3) |
| **Age >=65** | 18.1 (17.4-18.9) | | | 2.2 (1.3-3.0) | 6.1 (5.1-7.1) | 20.4 (19.5-21.2) |
| **Hispanic*** | 13.8 (12.4-15.3) | | | 11.4 (9.4-13.3) | 17.5 (15.3-19.7) | 13.7 (12.1-15.2) |
| **Non-Hispanic White** | 67.9 (65.9-69.9) | | | 72.7 (69.6-75.8) | 62.4 (59.5-65.3) | 67.9 (65.6-70.1) |
| **Non-Hispanic Black** | 11.1 (10.0-12.3) | | | 8.3 (6.8-9.9) | 12.8 (11.3-14.4) | 11.3 (10.0-12.6) |
| **Non-Hispanic Asian**** | 2.6 (2.2-3.0) | | | 2.9 (2.2-3.6) | 3.6 (2.9-4.4) | 2.5 (2.1-2.9) |
| **Non-Hispanic Others** | 4.5 (4.0-5.0) | | | 4.7 (3.2-6.1) | 3.7 (2.8-4.5) | 4.7 (4.0-5.3) |

**Table S5. Weighted Population Characteristics Overall and by CKM Syndrome Stages in US Adults**

Abbreviation: CKM, cardiovascular-kidney-metabolic syndrome.

* Hispanic represents Mexican American and other Hispanic individuals.

** non-Hispanic Asian participants were classified and oversampled since 2011.

|  | **Overall Population** | | | **CKM Stage 0** | | | **CKM Stage 1** | | | **CKM Stage 2** | | |
| --- | --- | --- | --- | --- | --- | --- | --- | --- | --- | --- | --- | --- |
| **Weighted Population** | 215238748 | | | 17111179 | | | 39430538 | | | 121793960 | | |
| **Variable** | **Weighted Prevalence % (95% CI)** | | | | | | | | | | | |
| **Overall** | 100.0 |  |  | 7.9 | 7.4 | 8.5 | 18.3 | 17.6 | 19.1 | 56.6 | 55.6 | 57.6 |
| **Men** | 48.7 | 48.1 | 49.4 | 36.0 | 33.1 | 39.0 | 51.3 | 49.2 | 53.3 | 50.7 | 49.8 | 51.7 |
| **Women** | 51.3 | 50.6 | 51.9 | 64.0 | 61.0 | 66.9 | 48.7 | 46.7 | 50.8 | 49.3 | 48.3 | 50.2 |
| **Age 18-24** | 9.2 | 8.6 | 9.8 | 22.9 | 20.5 | 25.4 | 13.2 | 11.8 | 14.5 | 5.7 | 5.1 | 6.4 |
| **Age 25-44** | 37.2 | 36.1 | 38.2 | 57.9 | 54.3 | 61.4 | 50.2 | 47.9 | 52.6 | 32.8 | 31.6 | 34.1 |
| **Age 45-64** | 35.6 | 34.6 | 36.5 | 17.0 | 14.4 | 19.6 | 30.5 | 28.4 | 32.6 | 41.1 | 39.9 | 42.3 |
| **Age >=65** | 18.1 | 17.3 | 18.8 | 2.2 | 1.3 | 3.0 | 6.1 | 5.0 | 7.1 | 20.3 | 19.5 | 21.2 |
| **Hispanic*** | 13.8 | 12.4 | 15.3 | 11.4 | 9.4 | 13.3 | 17.5 | 15.3 | 19.7 | 13.7 | 12.1 | 15.2 |
| **Non-Hispanic White** | 67.9 | 65.9 | 69.9 | 72.7 | 69.6 | 75.8 | 62.4 | 59.5 | 65.3 | 67.9 | 65.6 | 70.1 |
| **Non-Hispanic Black** | 11.1 | 10.0 | 12.3 | 8.4 | 6.8 | 9.9 | 12.9 | 11.3 | 14.4 | 11.3 | 10.0 | 12.6 |
| **Non-Hispanic Asian**** | 2.6 | 2.2 | 3.0 | 2.9 | 2.2 | 3.6 | 3.6 | 2.9 | 4.4 | 2.5 | 2.1 | 2.9 |
| **Non-Hispanic Others** | 4.5 | 4.0 | 5.0 | 4.7 | 3.2 | 6.1 | 3.6 | 2.8 | 4.5 | 4.7 | 4.1 | 5.3 |

**Table S6. Sensitivity Analysis with Reweighting for nonresponse**

* Hispanic represents Mexican American and other Hispanic individuals.

** non-Hispanic Asian participants were classified and oversampled since 2011.

|  | **Overall Population** | | | **CKM Stage 0** | | | **CKM Stage 1** | | | **CKM Stage 2** | | |
| --- | --- | --- | --- | --- | --- | --- | --- | --- | --- | --- | --- | --- |
| **Weighted Population** | 215238748 | | | 16948329 | | | 38773182 | | | 121722209 | | |
| **Variable** | **Weighted Prevalence % (95% CI)** | | | | | | | | | | | |
| **Overall** | 100.0 |  |  | 7.9 | 7.3 | 8.5 | 18.0 | 17.2 | 18.8 | 56.6 | 55.5 | 57.6 |
| **Women** | 51.3 | 50.6 | 51.9 | 64.1 | 61.1 | 67.1 | 48.9 | 46.8 | 51.0 | 49.0 | 48.1 | 50.0 |
| **Men** | 48.7 | 48.1 | 49.4 | 35.9 | 32.9 | 38.9 | 51.1 | 49.0 | 53.2 | 51.0 | 50.0 | 51.9 |
| **Age 18-24** | 9.2 | 8.6 | 9.8 | 23.3 | 20.8 | 25.8 | 13.3 | 12.0 | 14.7 | 5.5 | 4.9 | 6.2 |
| **Age 25-44** | 37.2 | 36.1 | 38.2 | 57.8 | 54.2 | 61.4 | 50.3 | 47.8 | 52.8 | 32.8 | 31.5 | 34.1 |
| **Age 45-64** | 35.6 | 34.6 | 36.5 | 16.9 | 14.3 | 19.5 | 30.5 | 28.3 | 32.7 | 41.2 | 40.0 | 42.5 |
| **Age >=65** | 18.1 | 17.3 | 18.8 | 2.0 | 1.1 | 2.8 | 5.9 | 4.8 | 6.9 | 20.4 | 19.6 | 21.3 |
| **Hispanic** | 13.8 | 12.4 | 15.3 | 11.5 | 9.5 | 13.5 | 17.3 | 15.1 | 19.5 | 13.7 | 12.2 | 15.3 |
| **Non-Hispanic White** | 67.9 | 65.9 | 69.9 | 72.7 | 69.5 | 75.9 | 62.7 | 59.9 | 65.6 | 67.8 | 65.5 | 70.1 |
| **Non-Hispanic Black** | 11.1 | 10.0 | 12.3 | 8.4 | 6.8 | 10.0 | 12.7 | 11.2 | 14.2 | 11.2 | 10.0 | 12.5 |
| **Non-Hispanic Asian** | 2.6 | 2.2 | 3.0 | 2.9 | 2.1 | 3.6 | 3.6 | 2.9 | 4.3 | 2.5 | 2.1 | 3.0 |
| **Non-Hispanic Others** | 4.5 | 4.0 | 5.0 | 4.5 | 3.1 | 6.0 | 3.7 | 2.8 | 4.6 | 4.7 | 4.0 | 5.3 |

**Table S7. Sensitivity Analysis with multivariate multiple Imputation**

* Hispanic represents Mexican American and other Hispanic individuals.

** non-Hispanic Asian participants were classified and oversampled since 2011.
